# Supplementary material for: Splice-Junction-Based Mapping of Alternative Isoforms in the Human Proteome
Source: Cell Rep. Author manuscript; Available in PMC 2020 Jan 15. (PMC6961840; doi:10.1016/j.celrep.2019.11.026)
Supplement: 2 [file NIHMS1546469-supplement-2.zip › DF1/PXD006675/RightAtrium/RightAtrium_10_PDLIM5_ANLSSSTGNVEDSFEGFR.pdf]

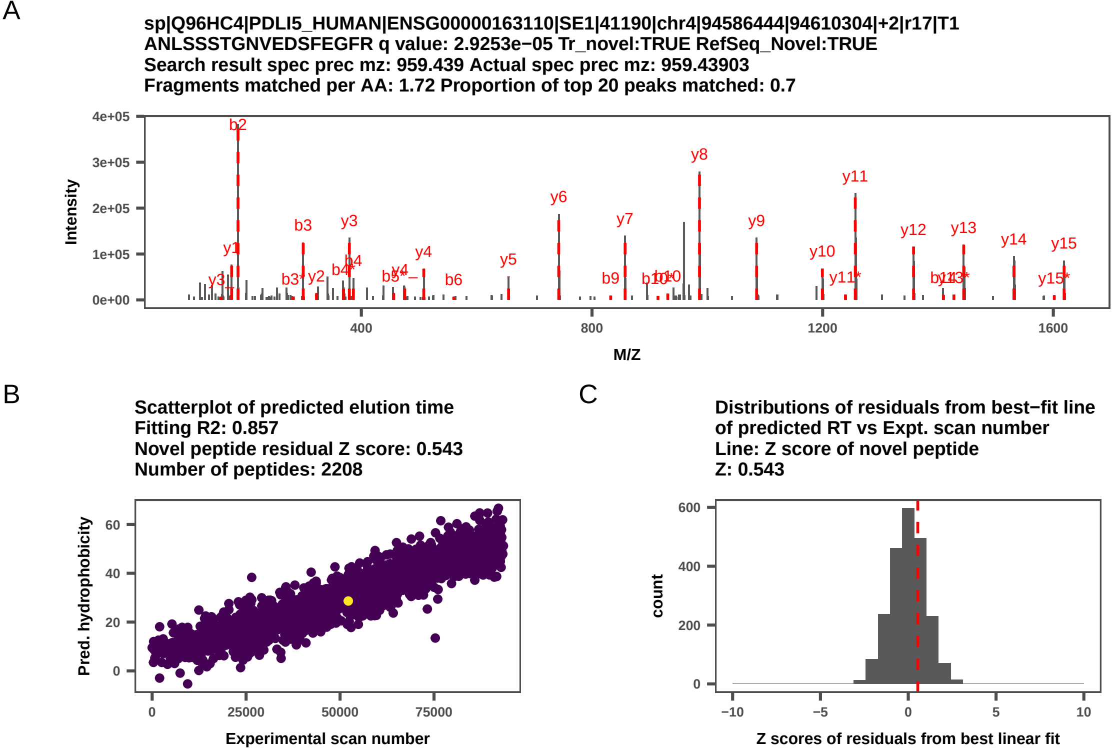
A
sp|Q96HC4|PDLI5_HUMAN|ENSG00000163110|SE1|41190|chr4|94586444|94610304|+2|r17|T1
ANLSSSTGNVEDSFEGFR q value: 2.9253e−05 Tr_novel:TRUE RefSeq_Novel:TRUE
Search result spec prec mz: 959.439 Actual spec prec mz: 959.43903
Fragments matched per AA: 1.72 Proportion of top 20 peaks matched: 0.7
Intensity
4e+05
3e+05
2e+05
1e+05
0e+00
b2
y1
y3*
b3
b3*
y2
y3
b4
b4*
b5*
y4
b6
y5
y6
b9
y7
b10*
y8
y9
y10
y11
y11*
y12
y13
y14
y15
y15*
400
800
1200
1600
M/Z
B
Scatterplot of predicted elution time
Fitting R2: 0.857
Novel peptide residual Z score: 0.543
Number of peptides: 2208
Pred. hydrophobicity
60
40
20
0
0
25000
50000
75000
Experimental scan number
C
Distributions of residuals from best−fit line
of predicted RT vs Expt. scan number
Line: Z score of novel peptide
Z: 0.543
count
600
400
200
0
−10
−5
0
5
10
Z scores of residuals from best linear fit
